# P-1717. Perception and Practice of Physicians' Antibiotic Use at Tertiary Military Hospitals in Bangladesh

**DOI:** 10.1093/ofid/ofae631.1882

**Published:** 2025-01-29

**Authors:** Syed Abul Hassan, Sharmi Saha, Md Golam Dostogir Harun, Md Shariful Amin Sumon, Ishrat Jahan, Md Mahabub Ul Anwar, Taufiq H Siddiquee

**Affiliations:** South Asia Field Epidemiology & Technology Network (Safetynet Bangladesh), Dhaka, Dhaka, Bangladesh; South Asia Field Epidemiology & Technology Network (Safetynet Bangladesh), Dhaka, Dhaka, Bangladesh; icddrb, Dhaka, Dhaka, Bangladesh; icddr,b, Dhaka, Dhaka, Bangladesh; Army HQ, Bangladesh Army, Dhaka, Dhaka, Bangladesh; Office of Health Affairs, West Virginia University, USA, Dhaka, Dhaka, Bangladesh; BRAC Healthcare, Dhaka, Dhaka, Bangladesh

## Abstract

**Background:**

Inappropriate use of antibiotics, which contributes to increased antimicrobial resistance (AMR), is common in healthcare settings across the globe, especially in low-resources settings. Antibiotics are widely and inappropriately used without adherence to international guidelines in Bangladesh, which may contribute significantly to the development AMR. Military hospitals are known for better compliance of health care system. We sought to investigate the perceptions of Physicians of Tertiary military hospitals in Bangladesh regarding appropriate antibiotic use and prescribing approach.

Physicians' AMR perception

**Figure:**
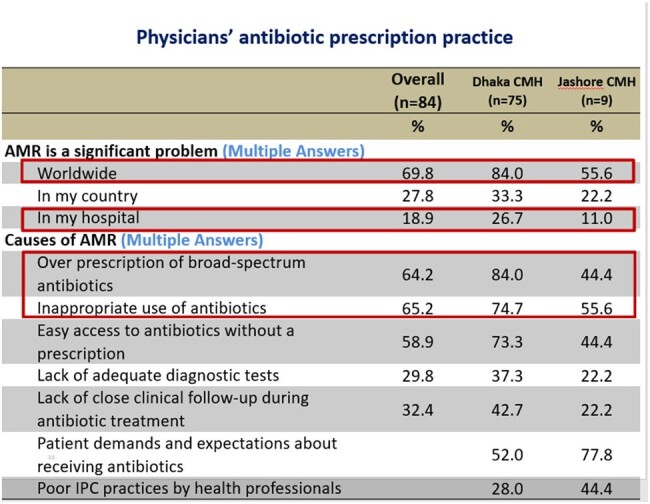

**Methods:**

We conducted a cross-sectional survey using a semi-structured questionnaire and data collected through face to face interview from 84 Military doctors of two major tertiary military hospitals of Bangladesh – Combined Military hospital (CMH) Dhaka (1650 bedded) and Jashore (500 bedded) between September 2020 and January 2021. Descriptive statistics were performed to analyze the data and for interpretation

Approach of physician to prescribing antibiotic

**Figure:**
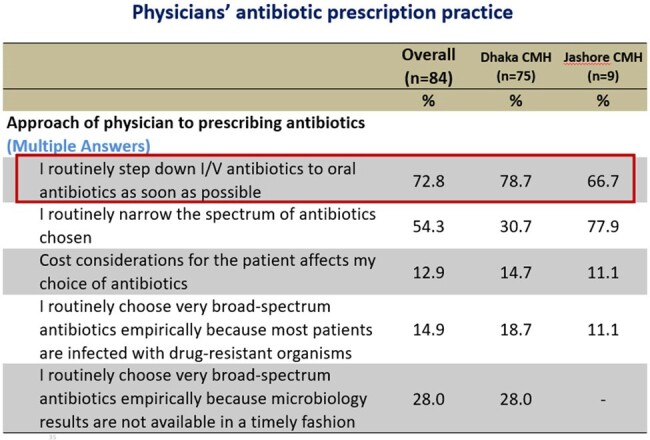

**Results:**

Out of 84 respondents, 70% of the physicians considered AMR is a significant concern worldwide, but only 26.7% Physician of Dhaka CMH and 11% of Jashore CMH considered it as a problem in their hospital. Two-third respondent (66%) considered that inappropriate use of antibiotic is the main cause of rising AMR, followed by Over prescription of broad-spectrum antibiotics by 62.2%, and Easy access to antibiotics without a prescription by 58.8%. Around 36.2% opined that poor IPC practices by health professionals are also important factor. Total 72.6% respondent ensure that they routinely step down I/V antibiotics to oral antibiotics as earliest, 13.4% physicians considered cost affordability of the patient affects their choice of antibiotics. Over half of the physicians (51.2%) use prophylactic antibiotic for 3 to 7 days after any surgical procedure of a clean-contaminated case without signs of infection, where only 7.3% doctor use single dose antibiotic prophylaxis.

Use of antibiotic prophylaxis in surgical procedure

**Figure:**
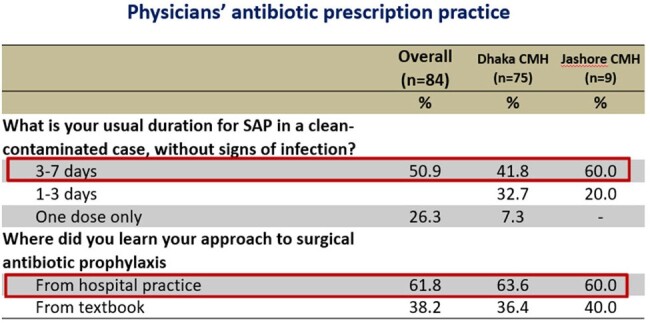

**Conclusion:**

Physicians' perception on AMR and rational antibiotic prescription were found fall of short of the standard. Strict compliance of antibiotic use and trade guideline, Introduction of formal antibiotic stewardship program in Bangladesh may be a useful intervention

**Disclosures:**

**All Authors**: No reported disclosures

